# Development of the nurse care assessment for in-hospital spinal cord injury rehabilitation

**DOI:** 10.1038/s41394-025-00702-4

**Published:** 2025-03-28

**Authors:** Frederik Skovbjerg, Stephanie Hilsløv Bøhm, Erhard Trillingsgaard Næss-Schmidt, Randi Kjær Steensgaard, Simon Svanborg Kjeldsen

**Affiliations:** 1 Spinal Cord Injury Centre of Western Denmark, Viborg Regional Hospital, Viborg, Denmark; 2 Department of Clinical Medicine, Faculty of Health, Aarhus University, Aarhus, Denmark; 3 Hammel Neurorehabilitation Centre and University Research Clinic, Hammel, Denmark; 4 The Danish Specialized Hospital for Polio and Accident Victims, Roedovre, Denmark

**Keywords:** Rehabilitation, Health care economics

## Abstract

**Study design:**

A development and reliability study.

**Objectives:**

To develop an assessment tool designed to categorize the care needs of inpatients with Spinal Cord Injuries.

**Setting:**

Spinal Cord Injury Centre of Western Denmark.

**Methods:**

Inspired by previous tools, NCA-SCI was refined through an iterative process with experienced clinicians. Content validity was established via consensus meetings and focus group interviews, resulting in 17 items across five categories: no/minor assistance, moderate assistance, severe nursing assistance, and unstable situations needing extensive nursing care. Face validity was ensured through iterative clinical feedback, and reliability was tested with four nurses scoring 36 patients.

**Results:**

Content validity and feedback led to a comprehensive, practical tool. Inter-rater reliability showed 81.4% agreement (Kappa = 0.69), while intra-rater reliability had 78.9% agreement (Kappa = 0.65), indicating moderate reliability.

**Conclusion:**

The NCA-SCI assesses nursing care needs in SCI rehabilitation, offering a practical tool with moderate reliability. The development of the NCA-SCI led to an easily usable tool for planning and coordinating daily care at a highly specialized unit.

## Background

Spinal cord injuries (SCI) implicate damage to the spinal cord resulting from trauma, disease, or degeneration, causing temporary or permanent changes in the functioning of people exposed [1, 2]. The injury disrupts the communication between the brain and the rest of the body, causing a loss of motor function, sensory function, autonomic function, or all below the level of the injury [3, 4]. Depending on the severity (Complete/incomplete) and the location of the injury, individuals may experience paralysis, altered sensation, and autonomic dysfunction [5]. SCIs significantly impact a person’s quality of life, requiring comprehensive medical, rehabilitative, and supportive care to manage the complex and multifaceted effects [6–8]. Rehabilitation after an SCI is complex due to the diverse needs related to the injury [9]. During in-hospital rehabilitation, the need for care varies between patients and over time due to multiple complications, different severity, and a range of physical and psychological conditions [10, 11]. Given the high turnover and shortage of nurses and healthcare workers in healthcare systems [12], it is essential to allocate resources and competencies according to patient needs to ensure patient safety and quality of care. Organizing and planning nursing and rehabilitation tasks in clinical wards is an ongoing, collaborative effort involving administrative and coordinating workload. This process is often time-consuming and challenging due to organizational constraints, workforce shortages, and competing responsibilities [13–16]. Therefore, it is vital to create tools that can consistently assess care needs and thereby assist in the daily coordination of care according to the needs of the admitted patients. Furthermore, identifying individual patient care needs can assist in long-term planning by enabling healthcare leaders to allocate resources more effectively during high-demand periods, when these periods can be systematically identified. Previous tools have been developed to provide a measure of the complexity of rehabilitation needs, care needs [17–21] or functioning. All these measures have in common that they are relatively time consuming and do not directly classify patients into e.g. minor, moderate or severe nursing needs that are easy to translate into daily clinical resource planning. Moreover, more specific interventions in relation to SCI rehabilitation are not necessarily contained in previous tools. Therefore, the purpose of this study was to develop an assessment tool designed to categorize the care needs of patients with SCI in an in-hospital rehabilitation setting.

## Methods

The Nurse Care Assessment for in-hospital Spinal Cord Injury Rehabilitation (NCA-SCI) was developed at the Spinal Cord Injury Centre of Western Denmark (SCIWDK). The center is a hospital-based 35-bed rehabilitation facility dedicated to spinal cord injured patients. SCI at the center is defined in accordance with the ASIA/ISCoS International Standards for Neurological Classification of Spinal Cord Injury (ISNCSCI). The items of the tool were inspired by previous tools developed by Turner-Stokes et al. [14, 15] and were tailored based on the expertise and input from clinicians at SCIWDK to meet the specific needs of patients undergoing rehabilitation.

### Participants

The group of developers included nurses with a median experience of 4 years working with SCI patients, ranging from 1–26 years. The 36 participants (patients) in the reliability study were selected based on their admission to the SCIWDK at the time of the study. They were not specifically asked to participate, as their data were accessed through their electronic medical records. This approach was approved as part of the study’s ethical guidelines. The participants included individuals with both complete and incomplete tetraplegia and paraplegia who were hospitalized for spinal cord injury rehabilitation.

### Development process

**Fig. 1:**
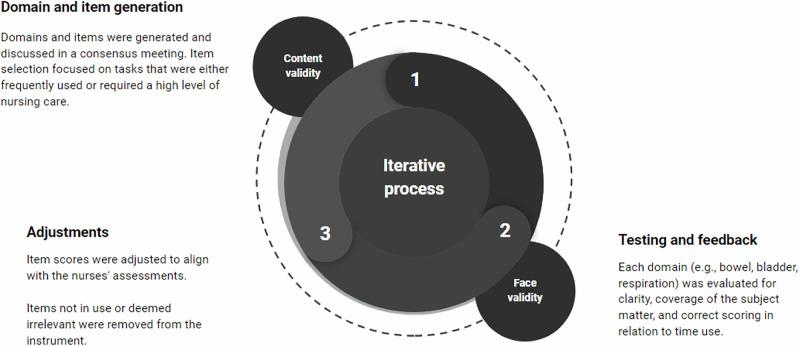
Overview of the development process.

The development of the NCA-SCI involved an iterative process with experienced clinicians, detailed in three main steps (see Fig. 1) and culminating in a reliability test. Throughout this process, we continuously worked on both content and face validity. In the first step, domains and items were generated and discussed during a consensus meeting with 12 experienced nurses, along with input from an occupational therapist. Item selection focused on tasks frequently used or requiring a high level of nursing care, with consideration given to items from previous instruments by Turner-Stokes et al. [22]. Consensus was required for item inclusion.

Similar to the assessment tool by Turner-Stokes et al., the NCA-SCI assessment involves evaluating each included item in relation to the individual patient’s condition and needs. Each item has its own specific scoring range, which reflects the intensity of care required for that particular aspect of rehabilitation. After scoring the individual items, a total score is calculated. This total score is then used to categorize patients into one of five groups: A, B, C, D, or E. Category E corresponds to patients with low or no care needs, while category A is designated for those requiring the highest level of care. Categories B, C, and D represent intermediate levels of care intensity. These categories are based on predefined cut-off values, which help classify the patients according to the overall intensity of care they require.

In the second step, the initial versions of the instrument were tested in clinical practice by eight nurses, followed by a focus group interview to further assess face and content validity. Based on the feedback, the instrument was adjusted accordingly. Each domain (e.g., bowel, bladder, respiration) was evaluated for clarity, coverage of the subject matter, and correct scoring in relation to time use.

In the third step, we refined the items and scores of version 10 through three iterations. Two nurses categorized 36 patients into categories A, B, C, D, and E using NCA-SCI, providing feedback on whether patients should belong to different categories. After each iteration, item scores were adjusted to align with the nurses’ assessments, and items not in use or deemed irrelevant were removed from the tool.

Finally, the inter-rater and intra-rater reliability of the instrument was tested with four nurses using a total of 36 patients. The cohort was split into two groups of 18 patients each, and each group was scored in categories A, B, C, D, and E by two nurses. A re-test was conducted 14 days later to assess consistency.

### Statistics

Inter and intra-rater reliability for categorizing A, B, C, D, and, E were reported between each pair of nurses and each nurse using Kappa statistics and confusion matrices.

## Results

We ensured content validity by discussing each item (e.g., bedwetting, incontinence, C-PAP) for its relevance to the nursing needs and identifying missing elements. This process involved narrowing down a large number of items to fewer, more significant ones. One nurse desired to use the tool to “show how many different tasks, were associated with the patients”, while another emphasized the importance of providing “a more nuanced picture” of patient differences. However, this conflicted with the need for the classification to be time-efficient and aligned with the primary aim. The feedback resulted in a first version including 22 items and 5 categories: no/minor assistance, moderate assistance, severe nursing assistance, and an unstable situation requiring extensive nursing assistance. Versions two through ten were adjusted based on feedback regarding item clarity, coverage, and time-related scoring. This iterative process continued until version 10, which was ready for step 3, securing the tool’s face validity.

### Agreement between the instrument and the nurses

The tool was iteratively refined over three rounds based on comparisons between the instrument’s scoring and the nurses’ assessments. In the first round, discrepancies were observed in 14 out of 31 patients, resulting in a 45% false scoring rate. After adjustments were made to the tool, the second round showed a significant improvement, with discrepancies reduced to 2 out of 24 patients (8% false scoring rate). By the third round, no discrepancies were observed across 31 patients, indicating that the tool had achieved complete agreement with the nurses’ assessments. After each iteration, the item scores were adjusted to achieve full agreement before proceeding to the next round. Text and response options for the individual items were simplified to minimize misinterpretation. The three iterations in step 3 reduced the total number of items to 17. The final NCA-SCI items are displayed in Table 1.

**Table 1:** Final items of the nurse care assessment for in-hospital spinal cord injury rehabilitation.

Item number	Item name	Options	Points
1	Respiratory support (BI-PAP, C-PAP, inhalation medicine, cough assist)	1	1
2	Teaching and guiding (Bladder, bowel, medicine, Activity of daily living, transfer)	1	1
3	Extensive psychological support	1	2
4	Registration of food and liquid intake	1	1
5	Observation of infection, blood pressure or pulse	1	2
6	Complication involving Fragmin or anti-embolic stockings	1	1
7	Complication involving Diabetes	1	1
8	Complication involving I.V. treatment	1	1
9	Complication involving severe pain affecting daily living	1	2
10	Complication involving severe spasticity affecting daily living	1	2
11	Isolation	1	3
12	Transfer (small transfer, using lift, lying support in bath)	3	1, 2, 3
13	Eating support (preparing eating, assistance when eating, tube feeding)	2	1, 2
14	Urinary tract complication (catheterization or other support, incontinent bladder > one time weekly)	2	1, 2
15	Bowel complications (klyx, stoma or using >30 min daily emptying bowel, incontinence or TAI)	2	1, 2
16	Support for activity of daily living (little to moderate support or help to almost everything)	2	1, 3
17	Wound care (level 1–2 or 3–4)	2	1, 2

*BI-PAP* BI-level positive airway pressure, *C-PAP* continuous positive airway pressure, *Fragmin* An anticoagulant medication, *I.V*. intravenous treatment, *TAI* transanal irrigation, a procedure used to manage bowel emptying, *Small transfers* refers to minor movements such as repositioning a patient in bed, moving from a bed to a chair, or short-distance transfers that require minimal assistance.

### Reliability

There was a total agreement of 81.4% between raters, with a Kappa value of 0.69 (SE = 0.08), *p* < 0.001, indicating a moderate level of agreement. For intra-rater reliability, there was a total agreement of 78.9%, with a Kappa value of 0.65 (SE = 0.08), *p* < 0.001, also indicating a moderate level of agreement. The inter- and intra-rater reliability of the 36 patients is reported in the confusion matrices in Table 2 and Table 3.

**Table 2:** Inter-rater reliability.

	E	D	C	B	A
**E**	4	1	0	0	0
**D**	3	34	4	0	0
**C**	0	2	15	3	0
**B**	0	0	0	4	0
**A**	0	0	0	0	0

**Table 3:** Intra-rater reliability.

	E	D	C	B	A
**E**	4	2	0	0	0
**D**	1	35	5	0	0
**C**	0	1	17	4	0
**B**	0	0	0	2	0
**A**	0	0	0	0	0

## Discussion

In the present study, we have developed and evaluated a tool for care intensity assessment at a highly specialized SCI rehabilitation unit. The tool was developed and evaluated through an iterative process involving three main steps. The steps involved securing feasibility, face validity, content validity, and testing the reliability. Through in-depth discussions among nurses, we sought to ensure that each item within the tool resonated with the core nursing needs of patients with SCI. The first part of the process revealed different perspectives e.g., some advocated for a comprehensive array of items to capture the nuanced complexities of patient care, while others emphasized the necessity of efficiency and alignment with the primary aim of the tool. Striking a balance between these competing demands was paramount, ultimately culminating in a refined version comprising 17 items categorized into five levels of nursing assistance.

To our knowledge, this study is the first to develop a SCI specific tool for identifying inpatient care needs in a hospitalized rehabilitation setting. There exists a variety of complexity classification tools within different genres of healthcare. We developed our tool with inspiration from the Rehabilitation Complexity Scale Extended (RCS-E) [15]. The RCS-E was initially designed to identify the clinical need for higher-level services as opposed to local services, especially within neurorehabilitation [22]. Since its initial development, the RCS-E has undergone further adjustments. While its discriminative purposes are highly relevant for referral and it includes important descriptive elements for rehabilitation, it primarily identifies the different professions and specialties that must be present. Therefore, it still overlooks specific areas of special care needs within the daily clinical practice of SCI rehabilitation. Whereas the RCS-E determines if the patient is in the appropriate department, our tool focuses on how to effectively distribute resources to meet patient needs. Additionally, our tool may also be further developed for referral purposes. The area of palliative care has also been subject to similar tools. A systematic review from 2021, investigated systems for the complexity of patient care needs, however, their findings were primarily targeted at identifying complexity in primary care and were used to address the care requirements of the individual for engaging appropriate resources and managing health service planning (e.g., for referral purposes similar to RCS-E) [23]. Other important classification tools include functional measures such as the Spinal Cord Independence Measure (SCIM) [21], which assesses the functional level of individuals and is routinely used within SCIWDK. However, the functional level does not necessarily translate into specific care needs and may not consider other critical components, such as psychological well-being and pain management. While a functional score provides valuable information for e.g. rehabilitation purposes, it may be insufficient for capturing the full spectrum of a patient’s needs. Therefore, care needs and functional scores can complement each other and could be considered for combined use in the future to provide a more comprehensive assessment of patients. A next step could be investigating the concurrent validity between the NCA-SCI and SCIM to determine how well these tools correlate and enhance the overall evaluation of patients’ needs.

Another area is the assessment of workload within clinical settings, as investigated in a review by Racey et al. [24]. Nursing workload can be affected in various ways, such as the time spent at the bedside with a patient, the competency level of the nurse, and the complexity of the care delivered; these measures describe another aspect of care needs [25]. With resource shortages, the workload and mental well-being of staff are at risk. In this context, our tool becomes essential for effectively balancing care needs and available resources. Further, the NCA-SCI tool can serve as a foundational framework for creating a common language among healthcare professionals when discussing patients and their care needs. By providing standardized criteria and categories, the tool can facilitate clear and consistent communication across multidisciplinary teams. This common language may ensure that all team members have a shared understanding of each patient’s condition, which is critical for coordinated care planning and delivery. A shared understanding may also enhance task-sharing among healthcare professionals. It may help delineate roles and responsibilities, ensuring tasks are delegated according to each professional’s expertise. By categorizing patient needs, the tool enables efficient allocation of routine tasks to support staff, allowing specialized clinicians to focus on complex care. It may also support flexibility and adaptability in dynamic healthcare settings, allowing for quick reassessments and adjustments to care plans.

### Strengths and limitations

Our quest for face validity underscored the iterative nature of tool refinement. Through multiple rounds of adjustments based on feedback from clinical settings, we fine-tuned the instrument to encompass relevant themes, enhance comprehensibility, and ensure accurate scoring of time use. This iterative process, spanning ten versions, exemplifies our commitment to refining the tool until it achieved initial agreement. Further, our exploration of agreement between the instrument and nurses yielded insights into the reliability and validity of our assessment tool. Initially, the high prevalence of false answers underscored the need for refinement. However, through meticulous adjustments to item scores and text, coupled with simplification efforts to minimize misinterpretation, subsequent rounds revealed significant improvements.

In the agreement between raters’ we only included four persons, however the results may still be indicative. Overall, the inter-and intra-rater reliability reached moderate levels, indicative of acceptable consistency in assessing care needs. The moderate Kappa values suggest that while the tool is generally reliable, there are some inconsistencies that could be addressed. Variability in raters’ interpretations and the subjective nature of the assessments might contribute to these discrepancies. Additionally, the 14-day interval between test occasions may have influenced the results, as it allows for potential real changes in patients’ conditions within an inpatient rehabilitation setting. This interval was selected to balance practical considerations, such as minimizing recall bias and accommodating clinical scheduling constraints. Further training and refinement of the tool could enhance reliability. In practice, establishing standardized procedures for patient assessments, including the frequency and the assessors involved, is crucial. Experienced assessors may enhance reliability over time, and more frequent assessments could improve validity and overall usability. However, these improvements should be balanced with available resources. When examining the confusion matrices for both inter- and intra-rater reliability, misclassifications primarily occur between adjacent categories. This pattern indicates a tendency toward ‘minor’ misclassifications, suggesting a low risk of incorrectly classifying an E as a B or vice versa, and thus a minimal risk of major clinical misinformation.

We only included nurses and one occupational therapist, which may have resulted in the inclusion of items and themes primarily relevant to these professions. As rehabilitation efforts highly rely on interdisciplinary teams, our approach could have benefited from including more professions in the development phase to create a more comprehensive and interdisciplinary tool. Lastly, the tool was specifically developed and tested within the context of SCIWDK and, hence, is currently applicable only within this context. However, we believe that the tool may be easily adjusted to use in other SCI rehabilitation wards and potentially also other medical specialties.

## Conclusions

In essence, our findings illuminate the dynamic interplay between theory and practice in the development and validation of nursing assessment tools. As we continue to refine and validate our tool, future research endeavors may delve deeper into its applicability across diverse patient populations and clinical settings. Another focus should be on developing an automated scoring system using data from electronic medical records to further streamline and improve care assessments and daily planning. By navigating the intricate landscape of care assessment, we aspire to enhance the quality of patient care and empower nursing professionals with robust tools for holistic patient evaluation and better planning in a daily clinical setting. The development of the NCA-SCI led to an easily usable tool for planning and coordinating daily care at a highly specialized unit, considering the complexity and current workload.

## Data Availability

The datasets generated during and/or analyzed during the current study are not publicly available due to the few individuals in some strata and the nature of the personal health data. Any questions regarding the dataset will be answered to the best of our ability by the corresponding author upon reasonable request.
